# Vitamin-K-antagonist phenprocoumon versus low-dose direct oral anticoagulants (DOACs) in patients with atrial fibrillation: a real-world analysis of German claims data

**DOI:** 10.1186/s12959-022-00389-9

**Published:** 2022-05-26

**Authors:** Lisette Warkentin, Susann Hueber, Barthold Deiters, Florian Klohn, Thomas Kühlein

**Affiliations:** 1 Friedrich-Alexander-Universität Erlangen-Nürnberg, Institute of General Practice, Universitätsklinikum Erlangen, Universitätsstraße, Erlangen, Germany; 2 GWQ ServicePlus AG, Gesellschaft für Wirtschaftlichkeit und Qualität bei Krankenkassen, Tersteegenstraße, Düsseldorf, Germany

**Keywords:** Direct oral anticoagulants, Phenprocoumon, Vitamin-K-antagonists, Atrial fibrillation, Low-dose therapy

## Abstract

**Background:**

For stroke prevention in patients with atrial fibrillation (AF), direct oral anticoagulants (DOACs) have been increasingly prescribed instead of vitamin-K-antagonists (VKA). For some patients a lower dosage of DOACs (ld-DOACs) is recommended. Ld-DOAC prescribing seems to be common, although previous studies did not show clear superiority of ld-DOACs over warfarin. In Germany, phenprocoumon is used almost exclusively as VKA. Randomized controlled trials comparing DOACs and phenprocoumon in the general population of patients with AF do not exist. Therefore, we aimed to compare ld-DOACs and phenprocoumon in a real-world setting in Germany.

**Methods:**

In a retrospective observational cohort study, claims data from a group of small to medium-sized health insurance companies were analysed. Risks for the outcomes thromboembolism, death and major bleeding were estimated by Cox regression. Out of 93,685 patients with atrial fibrillation and a first prescription of an oral anticoagulant, 20,179 receiving VKA and 21,724 ld-DOACs (29.6% of all DOAC patients) were included. For the sensitivity analysis phenprocoumon was compared to the five ld-DOAC groups (ld-apixaban, ld-dabigatran, ld-edoxaban, ld-rivaroxaban, and the composite of all ld-DOACs) after propensity-score matching.

**Results:**

Phenprocoumon was associated with statistically significant fewer thromboembolic events (HR = 1.29, 95% CI [1.13, 1.48], *p* < .001) and deaths (HR = 1.52, 95% CI [1.41, 1.63], *p* < .001) and a non-significant higher bleeding risk (HR = 0.89, 95% CI [0.79, 1.00], *p* = .051) than composite ld-DOAC. Regarding the subgroups, only patients with ld-apixaban had a statistically significant higher risk for thromboembolic events (HR = 1.42, 95% CI [1.21, 1.65], *p* < .001) and a lower bleeding risk (HR = 0.75, 95% CI [0.65, 0.86], *p* < .001). Ld-apixaban, ld-edoxaban, and ld-rivaroxaban were associated with a higher risk of death. The sensitivity analysis confirmed these associations.

**Conclusion:**

Phenprocoumon seems to be superior to ld-DOACs for patients with AF. As a hypothesis phenprocoumon might turn out to be the wiser choice for high-risk patients with AF as compared to ld-DOACs, especially regarding thromboembolic events and death. Therefore, RCTs comparing ld-DOACs with phenprocoumon are needed.

**Supplementary Information:**

The online version contains supplementary material available at 10.1186/s12959-022-00389-9.

## Background

Atrial fibrillation (AF) is the most common arrhythmia [1]. It is accompanied by an increased risk of thromboembolic stroke [1, 2]. For most patients with AF, oral anticoagulation (OAC) is thus recommended for stroke prevention [3]. Vitamin-K-antagonists (VKA) have been the standard substances for OAC for a long time. Starting with the drug approval of the first direct oral anticoagulant (DOAC) dabigatran etexilate [4], a trend towards prescribing DOACs instead of VKA gained momentum. At this current time four different DOACs are approved in Germany: dabigatran, rivaroxaban, apixaban, and edoxaban. The pivotal randomized controlled trials (RCT) partially showed statistically significant but small risk reductions or at least non-inferiority regarding the outcomes stroke/systemic embolism, and major bleeding as compared to warfarin [5–8].

For patients with a higher risk of bleeding due to patient-specific criteria like severely impaired renal function, old age, or reduced body-weight, the European Medicines Agency (EMA) and the Drug Commission of the German Medical Association (AkdÄ*)* recommend DOACs to be prescribed in lower dose [3, 9–12]. Low-dose DOAC (ld-DOAC) therapy is common internationally. In the ORBIT-AF II registry (USA), 16% of patients with DOACs received a reduced dose [13]. Higher rates of ld-DOACs were reported in Denmark and Germany, ranging from 32 to 52% [14–17]. In a Japanese single-centre cohort study, the ld-DOAC cohort included as many as 56% of patients [18]. Only for dabigatran and edoxaban effectiveness and safety of ld-DOAC therapy was compared to warfarin in pivotal RCTs [6, 7]. A reduced dose seemed to be partially associated with a higher risk for thromboembolic events and a lower risk for bleeding. In a cohort study from Denmark comparing ld-DOACs with warfarin, event rates of ischemic stroke or systemic embolism did not differ [19]. Bleeding rate was significantly lower only with dabigatran. A real-world study by Hohnloser et al. revealed that the risk for ischemic stroke with ld-DOACs was similar to phenprocoumon but the bleeding risk partially decreased with ld-DOACs [14]. Although in Germany phenprocoumon is used almost exclusively as VKA, to our knowledge, there are no RCTs comparing phenprocoumon and DOACs in the general population of patients with AF. Phenprocoumon differs in its pharmacokinetic properties as compared to warfarin, as it has for example a longer half-life [20]. Studies have shown that time in therapeutic range (TTR) in Germany with phenprocoumon is better than in the RCTs comparing warfarin to DOACs elsewhere [5–8, 21, 22]. Therefore, transferring the results of the comparison of DOACs or even ld-DOACs with warfarin to phenprocoumon does not seem appropriate.

The aim of this study was to add to the current evidence - with ambiguous results - by comparing the effectiveness and safety of OAC for patients with AF treated with ld-DOACs as opposed to phenprocoumon in a real-life setting. To our knowledge, this is the first empirical study comparing phenprocoumon with all four DOACs approved in Germany (apixaban, edoxaban, dabigatran, rivaroxaban) in reduced dosage.

## Method

A retrospective observational cohort study using German claims data of several company health insurance funds was conducted. Routine health care data were provided and analysed by the Corporation for Efficiency and Quality in Health Insurance (GWQ ServicePlus AG, Gesellschaft für Wirtschaftlichkeit und Qualität bei Krankenkassen: FK, BD). It is owned by a group of health insurance companies comprising up to 10.5 million insurants in Germany. The reporting of the study is based on the German GPS (Good Practice Secondary Data Analysis) [23] and the RECORD (Reporting of studies conducted using observational routinely-collected health data) statement [24].

### Data and study population

**Fig. 1:**
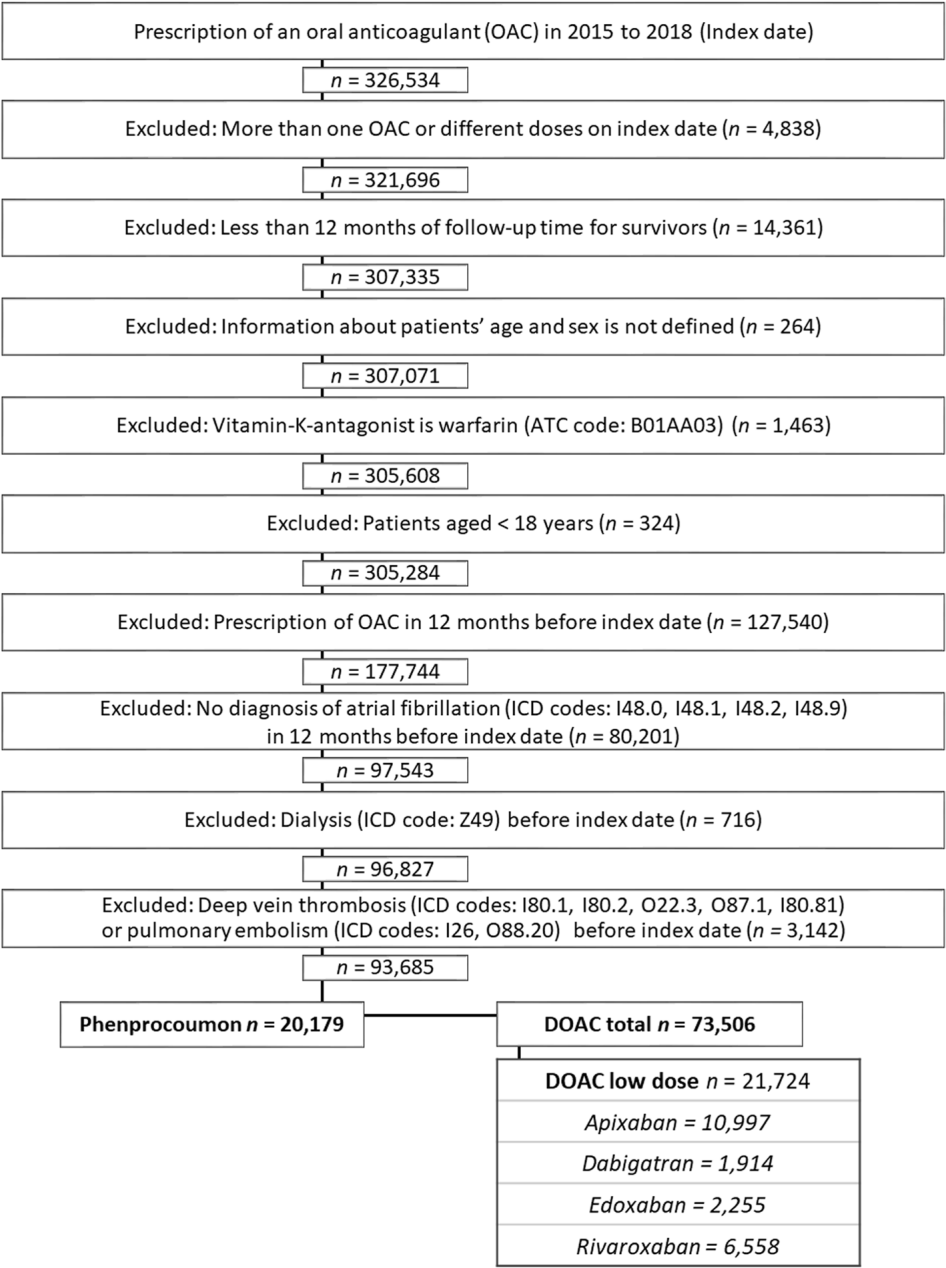
Data selection process. Index date is defined as the date of first prescription of an oral anticoagulant

The dataset included information from outpatient and inpatient care (age, sex, diagnoses, and medications). Data from the years 2014 to 2019 were analysed. Claims data that could not be linked to patients due to bad coding was corrected as far as possible with an internal mapping algorithm. To achieve a dataset of patients with the possibility of at least one year of follow-up with continuous insurance status, patients with a first prescription of OAC in 2015 to 2018, defined as index date, were included. Furthermore, patients had to have at least one in- or outpatient diagnosis of AF and no OAC prescription during the pre-index period of 12 months, and at least 12 months of follow-up time after the index date, to be included. In case of death during the observation period there was no minimum follow-up time. For the survivors, continuous insurance status was defined as being insured in the beginning and end of the observation period and having at least one observable insurance day in each observable quarter. Exclusion criteria were receiving more than one oral anticoagulant, receiving DOACs in both low and standard dose or receiving warfarin as VKA on index date. Other VKAs were not prescribed. Datasets with an undefined age and/or sex, age younger than 18 years, and dialysis were also excluded. Patients with pulmonary embolism and/or deep vein thrombosis during the pre-index period as competing indication for OAC were excluded from the sample. Data of patients were selected as shown in Fig. 1.

From the DOAC sample only patients with ld-DOACs were considered. Ld-DOAC treatment was defined as a dose smaller than the standard dose as suggested for prevention of thromboembolism in AF by the summaries of product characteristics (SmPCs) of the respective DOAC (standard dose: apixaban: 2 × 5 mg/day, edoxaban: 60 mg/day, dabigatran 2 × 150 mg/day, rivaroxaban 20 mg/day) [9–12]. Dosing was operated by the pharmacy-central-number (PZN = identification number for pharmaceutical products in Germany) (Table S1, additional file 1) [3].

### Outcome measures

In line with the other real-world studies, the observation period was chosen to be 12 months beginning with the date of first prescription [14, 15]. Effectiveness outcomes were hospitalization due to thromboembolic events, including ischemic stroke, non-specified stroke, transient ischemic attack, and mesenteric ischemia. Another outcome was death of any cause (death coded as reason for deregistration from health insurance). Safety outcomes were major bleedings defined as hospitalizations due to bleeding in critical areas or organs, like intracranial bleeding, and other bleedings which led to blood transfusion. The choice was made according to the criteria of the International Society on Thrombosis and Haemostasis (ISTH) (ICD-10-codes in Table S2, additional file 1) [25].

### Statistical analysis

Comparisons were made between phenprocoumon and five ld-DOAC groups (ld-apixaban, ld-dabigatran, ld-edoxaban, ld-rivaroxaban and the composite of all ld-DOACs). Calculating event rates, only the first event per patient was considered for each outcome. Rates were calculated per 100 patient years. Event rates and Cox regression were censored for death and switch in medication and/or dose.

Cox regression models were applied to estimate effectiveness and safety of treatments with adjusted cause specific hazard ratios. Risk adjustment was done based on the following pre-treatment control variables to reduce confounding: (1) age and sex; (2) comorbidities, e.g. arterial hypertension, cachexia, and renal impairment; (3) comedication, e.g. antiarrhythmic, antihypertensive medication (ICD-10-codes/ATC-codes in Table S3, additional file 1); (4) CHA_2_DS_2_-VASc-Scores, calculated based on the dataset (sex not included, as it is considered separately); (5) Charlson Comorbidity Index (CCI, [26]); (6) effectiveness and safety outcomes that occurred before the index date; (7) dummy variables for each year/quarter of the index dates. As the CHA_2_DS_2_-VASc -Score has similar predictive performance as the widely used HASBLED and other predictive scores, we refrained from adjusting to another bleeding risk score [27]. Multicollinearity tests were applied to test whether the treatment effect could be properly distinguished from the confounders.

A sensitivity analysis was carried out using propensity score matching (PSM). Propensity scores were estimated using the same explanatory variables as in the Cox regressions. A 1:1 nearest neighbour matching without replacement was performed under the constraint that maximum standardized mean difference between the groups had to be < 0.1 for all confounders. Year and time dummies were excluded when estimating the propensity scores in order to achieve a comparable treatment and control group, as the composition of VKA/DOACs changed over time. Cohort-pairs (*n* = 5) were formed for all ld-DOAC groups using logistic regression with ld-DOAC patients used as binary dependent variables in the first stage of the matching process. Sample sizes after PSM, number of patients for whom no matching partner was found, and baseline characteristics after PSM are depicted in Table S4, additional file 1.

After PSM, a two-sample test for equality of proportions with continuity correction was performed for the outcomes thromboembolic events, death, and bleeding.

To counteract the problem of multiple testing *p*-values were adjusted with Bonferroni correction (*n =* 15 tests) separately for Cox regression and sensitivity analysis. An adjusted two-sided *p*-value < .003 was considered significant. Statistical analyses were performed using R Statistical Software (version 3.6.1) [28–30].

## Results

### Baseline characteristics and unadjusted outcome rates

In total, 73,506 patients received DOACs. Of the latter 21,724 (29.6%) received ld-DOACs. After excluding patients with DOAC in standard dose (*n* = 51,782), data from 41,903 patients were analysed, of which 20,179 received phenprocoumon. The baseline characteristics are reported as proportion or mean with standard deviation in Table 1. Patients in the ld-DOAC cohort were more likely to be female, were older, had more comorbidities, and a higher CHA_2_DS_2_-VASc-Score.

**Table 1:** Baseline characteristics of phenprocoumon, composite low-dose DOAC (ld-DOAC) cohort and single ld-DOAC cohorts

	Phenprocoumon	ld-DOAC	ld-Apixaban	ld-Dabigatran	ld-Edoxaban	ld-Rivaroxaban
*n*	20,179	21,724	10,997	1914	2255	6558
Baseline characteristics: mean (SD)						
Age	74.81 (10.11)	79.51 (9.67)	81.48 (8.70)	76.5 (10.36)	79.96 (9.19)	76.94 (10.34)
Female persons (%)	41.70	49.77	53.19	43.78	53.48	44.50
CCI	3.03 (2.08)	3.58 (2.15)	3.77 (2.14)	3.2 (2.1)	3.5 (2.18)	3.39 (2.13)
CHA_2_DS_2_-VASc-Score (without gender)	3.7 (1.59)	4.21 (1.56)	4.41 (1.49)	4.06 (1.65)	4.05 (1.53)	3.98 (1.61)
Prescriptions in addition to OAC	9.81 (5.28)	10.81 (5.55)	11.13 (5.55)	9.89 (5.18)	10.37 (5.46)	10.7 (5.63)
Comorbidities: Proportion of patients with … (%)						
Acute renal impairment	6.49	9.59	11.34	4.96	9.62	7.99
Moderate chronic renal impairment	22.56	32.57	35.37	21.06	36.72	29.80
Severe chronic renal impairment	6.00	6.44	8.07	1.57	7.36	4.82
Renal impairment (total)	33.80	45.59	49.65	29.83	49.53	42.04
Dementia	8.66	17.90	21.14	13.06	16.54	14.33
Thrombosis	3.68	4.00	3.92	2.72	3.28	4.77
Arterial hypertension	89.55	91.25	92.37	89.92	89.76	90.29
Diabetes	36.58	39.97	40.27	35.32	39.96	40.85
Smoker	8.22	7.14	6.27	8.15	6.16	8.65
Alcohol abuse	3.03	3.42	3.19	3.19	3.41	3.86
Myocardial infarction	8.80	11.84	10.78	13.48	7.80	14.53
Stroke	4.20	5.61	6.29	7.84	3.99	4.36
Atherosclerosis	19.68	21.69	22.23	20.38	21.64	21.20
Cancer	21.50	24.73	25.84	21.79	26.03	23.28
Liver disease	18.48	18.11	17.77	17.29	20.22	18.19
Cachexia	0.69	1.95	2.31	0.84	1.86	1.69
Adiposity	28.09	24.36	23.19	22.15	23.73	27.19
Orthopaedic implant	3.49	8.83	9.04	5.22	11.49	8.63
Comedication: Proportion of patients with … (%)						
Antihypertensive medication	7.45	7.92	8.25	6.74	9.00	7.35
Heparin	26.77	10.13	9.97	9.67	10.38	10.46
Diuretics	54.96	59.63	64.05	48.69	57.34	56.19
Antiarrhythmic med.	87.76	86.97	87.59	85.16	85.68	86.92
NSAIDs	32.88	32.79	31.45	34.74	30.95	35.10
Antiplatelet therapy	26.05	36.83	36.72	36.31	30.64	39.28
Lipid lowering medication	47.88	48.15	47.48	54.08	45.19	48.57
Anti-ulcer therapy	47.10	53.65	55.26	49.01	49.36	53.80
Cardiac glycosides	11.44	9.91	10.76	7.94	8.65	9.50
Oral corticosteroids	12.74	14.51	14.45	14.37	14.01	14.84
Antipsychotic medication	4.39	9.26	10.65	7.68	8.38	7.69
Inpatient diagnosis before index date … (%)						
Thromboembolic event	5.80	11.91	14.28	20.79	7.14	6.98
Bleeding	2.45	3.05	3.58	3.08	2.08	2.47
Outpatient diagnosis before index date … (%)						
Thromboembolic event	10.32	13.97	15.26	19.64	11.13	11.13
Bleeding (no blood transfusion)	1.58	2.09	2.38	1.99	1.95	1.69

Observed crude event rates before matching indicated a higher risk for all outcomes for the composite ld-DOAC cohort. This was consistent regarding the single ld-DOAC subgroup analyses, with exception to bleeding in patients taking dabigatran (event rates per 100 patient-years: phenprocoumon = 4.05, ld-apixaban = 4.26, ld-dabigatran = 3.52, ld-edoxaban = 4.84, ld-rivaroxaban = 5.48) (Table 2). The mean follow-up times are depicted in Table S5, additional file 1.

**Table 2:** Event rates per 100 patient-years (py) for the outcomes thromboembolic events, death and bleeding before and after propensity-score matching

	**Phenprocoumon**		**Ld-DOAC**	
**Event rate per 100 py**	**Before matching**	**After matching**	**Before matching**	**After matching**
***n***	20,179	14,818	21,724	14,818
Thromboembolic events	2.53	2.83	4.42	3.93
Deceased	7.45	8.57	18.51	15.01
Bleeding	4.05	4.53	4.61	4.22
	**Phenprocoumon**		**Ld-Apixaban**	
**Event rate per 100 py**	**Before matching**	**After matching**	**Before matching**	**After matching**
***n***	20,179	8991	10,997	8991
Thromboembolic events	2.53	3.19	5.18	4.87
Deceased	7.45	11.37	22.55	19.32
Bleeding	4.05	5.31	4.26	4.12
	**Phenprocoumon**		**Ld-Dabigatran**	
**Event rate per 100 py**	**Before matching**	**After matching**	**Before matching**	**After matching**
***n***	20,179	1908	1914	1908
Thromboembolic events	2.53	3.09	3.59	3.59
Deceased	7.45	9.42	9.49	9.23
Bleeding	4.05	3.66	3.52	3.52
	**Phenprocoumon**		**Ld-Edoxaban**	
**Event rate per 100 py**	**Before matching**	**After matching**	**Before matching**	**After matching**
***n***	20,179	2235	2255	2235
Thromboembolic events	2.53	3.23	3.74	3.78
Deceased	7.45	11.34	16.07	15.76
Bleeding	4.05	4.98	4.84	4.82
	**Phenprocoumon**		**Ld-Rivaroxaban**	
**Event rate per 100 py**	**Before matching**	**After matching**	**Before matching**	**After matching**
***n***	20,179	6478	6558	6478
Thromboembolic events	2.53	2.90	3.60	3.55
Deceased	7.45	9.51	14.96	14.67
Bleeding	4.05	4.83	5.48	5.35

Event rates before and after propensity-score matching in phenprocoumon cohort, composite low-dose DOAC (ld-DOAC) cohort and single ld-DOAC cohorts (ld-apixaban, ld-dabigatran, ld-edoxaban and ld-rivaroxaban)

### Cox regression analysis

#### Safety and effectiveness for the composite ld-DOAC cohort

Results showed statistically significant fewer thromboembolic events and deaths with phenprocoumon as compared to ld-DOACs. There was a non-significant trend of fewer bleedings with composite ld-DOACs (thromboembolic events: HR = 1.29, 95% CI [1.13, 1.48], *p* < .001; death: HR = 1.52, 95% CI [1.41, 1.63], *p* < .001; bleeding: HR = 0.89, 95% CI [0.79, 1.00], *p* = .051).

#### Safety and effectiveness in single ld-DOAC subgroups

Regarding the single ld-DOAC subgroups, the effect of fewer thromboembolic events with phenprocoumon was only statistically significant in the ld-apixaban cohort. In the other cohorts, risks did not differ significantly from phenprocoumon (phenprocoumon vs. ld-apixaban: HR = 1.42, 95% CI [1.21, 1.65], *p* < .001).

All subgroup cohorts except ld-dabigatran were associated with a higher risk of death than phenprocoumon (ld-apixaban: HR = 1.63, 95% CI [1.50, 1.76], *p* < .001; ld-dabigatran: HR = 1.12, 95% CI [0.94, 1.34], *p* = .193; ld-edoxaban: HR = 1.40, 95% CI [1.22, 1.60], *p* < .001; ld-rivaroxaban: HR = 1.45, 95% CI [1.32, 1.59], *p* < .001).

**Fig. 2:**
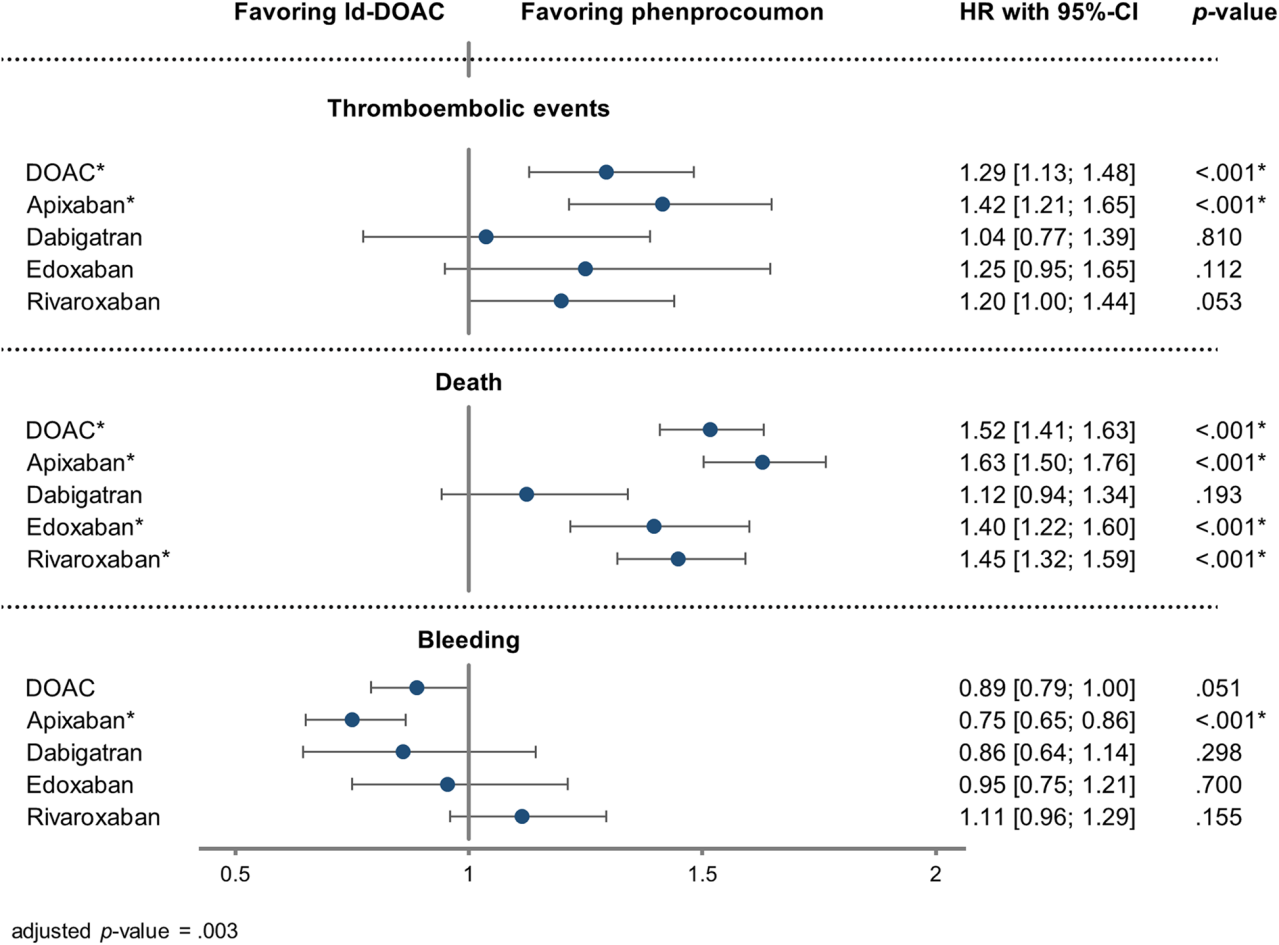
Cox proportional hazard regression model for the comparison of low-dose DOAC versus phenprocoumon. Adjusted hazard ratios with 95%-confidence interval and *p*-value adjusted with Bonferroni correction (adjusted *p*-value < .003, *n* = 15)

A statistically significant lower bleeding risk was shown only for the ld-apixaban cohort. In the ld-dabigatran and ld-edoxaban cohorts a slight tendency towards a lower bleeding risk was shown. For the ld-rivaroxaban cohort the association was reversed (ld-apixaban: HR = 0.75, 95% CI [0.65, 0.86], *p* < .001; ld-dabigatran: HR = 0.86, 95% CI [0.64, 1.14], *p* = .298, ld-edoxaban: HR = 0.95, 95% CI [0.75, 1.21], *p* = .700; ld-rivaroxaban: HR = 1.11, 95% CI [0.96, 1.29], *p* = .155). The results are depicted in Fig. 2. Hazard ratios for all covariates are depicted in Fig. S1, additional file 1.

### Sensitivity analysis

Results of the comparison of phenprocoumon and ld-DOACs regarding effectiveness and safety with Cox regression models showed consistency in analysis after PSM (Table 3). The outcome death was associated with the highest absolute risk increases with ld-DOAC subgroups compared to phenprocoumon ranging from 2.8% in the ld-rivaroxaban cohort to 5.7% in the ld-apixaban cohort. Regarding the outcome major bleeding, ld-apixaban was associated with a statistically significant absolute risk reduction (ARR = 1.2, 95% CI [0.6, 1.8%]). The event rates per 100 patient-years after matching are shown in Table 2.

**Table 3:** Results of analysis of effectiveness and safety of low-dose DOAC (ld-DOAC) versus phenprocoumon

	Propensity Score Matching				Cox-Regression	
Treatment	RRR	ARR [95%-CI]	NNT	*p*-value	HR [95% CI]	*p*-value
**Thromboembolic events**						
**Ld-DOAC**	**−24.0%**	**−0.6% [− 1.0%; − 0.2%]**	**− 172**	.002*	**1.29 [1.13; 1.48]**	< .001*
**Ld-Apixaban**	**−42.1%**	**−1.1% [− 1.7%; − 0.6%]**	**−89**	< .001*	**1.42 [1.21; 1.65]**	< .001*
Ld-Dabigatran	−6.0%	−0.2% [− 1.2%; 0.9%]	− 636	0.842	1.04 [0.77; 1.39]	.810
Ld-Edoxaban	−10.0%	−0.3% [− 1.3%; 0.7%]	− 373	0.651	1.25 [0.95; 1.65]	.112
Ld-Rivaroxaban	−7.0%	−0.2% [− 0.7%; 0.4%]	− 589	0.575	1.20 [1.00; 1.44]	.053
**Death**						
**Ld-DOAC**	**−56.9%**	**−4.2% [− 4.9%; −3.5%]**	**−24**	< .001*	**1.52 [1.41; 1.63]**	< .001*
**Ld-Apixaban**	**−59.0%**	**−5.7% [−6.6%; − 4.7%]**	**−18**	< .001*	**1.63 [1.50; 1.76]**	< .001*
Ld-Dabigatran	10.4%	0.8% [−0.9%; 2.6%]	119	0.361	1.12 [0.94; 1.34]	.193
**Ld-Edoxaban**	**−31.0%**	**− 3.0% [−4.8%; −1.1%]**	**−34**	.002*	**1.40 [1.22; 1.60]**	< .001*
**Ld-Rivaroxaban**	**−34.5%**	**−2.8% [− 3.8%; −1.8%]**	**−36**	< .001*	**1.45 [1.32; 1.59]**	< .001*
**Bleeding**						
Ld-DOAC	16.7%	0.6% [0.2%; 1.1%]	156	0.003	0.89 [0.79; 1.00]	.051
**Ld-Apixaban**	**27.2%**	**1.2% [0.6%; 1.8%]**	**83**	< .001*	**0.75 [0.65; 0.86]**	< .001*
Ld-Dabigatran	11.9%	0.4% [−0.8%; 1.5%]	273	0.563	0.86 [0.64; 1.14]	.298
Ld-Edoxaban	8.7%	0.4% [−0.8%; 1.5%]	279	0.590	0.95 [0.75; 1.21]	.700
Ld-Rivaroxaban	3.8%	0.2% [−0.5%; 0.8%]	648	0.685	1.11 [0.96; 1.29]	.155

Results of comparison after propensity score matching and the significant results of the Cox regression models. Bold text highlights statistically significant results. Adjustment of *p*-value with Bonferroni correction

*RRR* Relative risk reduction, *ARR* Absolute risk reduction, *NNT* Number needed to treat

*adjusted *p*-value = .003

## Discussion

The analysis of routine health-care data revealed a small but statistically significant higher risk for thromboembolic events and death for patients with ld-DOAC as compared to phenprocoumon. A non-significant association towards a lower severe bleeding risk in patients with ld-DOAC compared to phenprocoumon could be seen. Regarding the single ld-DOAC subgroups, ld-apixaban has on the one hand a small disadvantage in effectiveness and on the other hand a small advantage concerning major bleeding. All ld-DOACs but ld-dabigatran were associated with a significantly higher risk of death.

Severe renal impairment is one of the main indications for dose reduction of DOACs [3]. The rate of ld-DOAC patients with renal impairment in our study was less than 50%. Although quality of coding renal impairment in claims data is known to be low, this still might indicate that a noticeable part could be underdosed with ld-DOAC. Previous studies showed that many patients (22 to 57%) with ld-DOAC do not meet the criteria for reduced dose [13, 16, 18, 31]. Assumingly, prescribing a lower dose than recommended might be induced by the intention to protect the patient, as inadequate ld-DOAC is often prescribed in elderly patients at a higher risk for bleeding and stroke [16, 18, 31, 32]. In the case of VKA therapy, patients at risk would possibly receive a close INR-monitoring to prevent overdosing or a dose targeted at the lower border of INR, as low-intensity VKA-therapy was shown to be as efficient and safer as standard VKA-therapy in a recent meta-analysis [33]. However, a routine monitoring for DOAC therapy is not established. Triggered by caution and uncertainty this could lead to an inappropriate dose reduction in patients with an assumed higher bleeding risk. Previous studies demonstrated that this could harm the patient: A higher all-cause mortality and a similar risk for stroke or systemic embolism with inadequate compared to adequate DOAC dosing [31] and a 2.5-fold increase of risk for thromboembolic events compared to VKA were recently reported [34]. Hence, ld-DOAC should be prescribed according to the recommendations and guidelines.

Inadequate and non-recommended prescribing of ld-DOACs could be a factor that influenced our results. In our analysis we combined patients with adequate and inadequate ld-DOAC therapy. Combined, the only statistically significant beneficial effect of ld-DOAC was seen for bleeding in patients with ld-apixaban. Thus, phenprocoumon seems to be superior as compared to ld-DOACs as prescribed today in Germany. However, the results might be different if ld-DOACs were prescribed only for patients meeting the guideline criteria. As laboratory parameters of for example renal function and information on patients’ weight are not part of our data, we could not but conflate data of patients with adequate and inadequate ld-DOACs.

### Comparison of the results to the existing literature

To our knowledge, there is no RCT comparing efficacy and safety of DOACs and phenprocoumon in patients with AF except for a few studies with focus on special comorbidities like end-stage kidney disease or special situations like catheter ablation [35–39]. For most of them the results are not yet published. The major RCTs compared DOACs with warfarin. To conduct an appropriate comparison of our results to the existing literature, we first focus on two other real-world studies comparing ld-DOACs with phenprocoumon before we discuss why the results of our study might differ from those of RCTs conducted with warfarin.

### Comparison to other real-world studies with ld-DOACs and phenprocoumon

#### Effectiveness

As in our study, a significantly higher risk for thromboembolic events in patients with ld-DOACs than with phenprocoumon was also shown by Mueller et al. [15]. In the study of Hohnloser et al. at least ld-apixaban was associated with an even lower risk for the composite outcome stroke/systemic embolism [14]. However, in terms of ischemic stroke, the differences between the single ld-DOACs and phenprocoumon were not statistically significant. In contrast to our study and the one by Mueller et al., Hohnloser et al.’s study included haemorrhagic stroke to the composite outcome stroke and systemic embolism.

#### Bleeding risk

Contrary to our results a benefit of phenprocoumon over composite ld-DOACs regarding severe bleeding risk was shown by Mueller et al. [15]. Their composite DOAC cohort, however, comprised mainly patients with rivaroxaban. Hohnloser et al. reported a beneficial effect of ld-apixaban, also shown by our results [14]. In contrast to our results they also found a lower major bleeding risk with ld-dabigatran; however, in terms of any bleeding, intracranial bleeding, and gastrointestinal bleeding no significant differences were shown. The risk of intracranial bleeding was lower than the risk of gastrointestinal bleeding with ld-apixaban, ld-dabigatran, and ld-rivaroxaban. Our study cannot validate this effect as we did not differentiate between different types of bleeding. A possible explanation for the lower bleeding risk of apixaban in comparison to the other DOACs might be found in pharmacokinetics [40, 41]. Apixaban is the only factor Xa inhibitor that is administered twice daily. However, the exact reason is not known.

#### Risk of death

In line with our results, Hohnloser et al. found a higher risk of death with ld-rivaroxaban and ld-apixaban, although the trend for ld-apixaban was not statistically significant [14]. Ld-edoxaban was only analysed in our study and was associated with a higher risk of death as well. The cause of seemingly increased risk of death for patients with ld-DOAC remains uncertain in our data. Residual confounding leading to the seeming appearance of an association between risk of death and ld-DOAC therapy has to be taken into consideration as a partial explanation.

### Why the results of our study might differ from those of RCTs conducted with warfarin

Only in the pivotal RCTs for dabigatran and edoxaban DOACs were analysed in reduced dosage separately [6, 7]. Those results partially differed from our results. Specific methodological characteristics of RCTs and real-world studies might trigger discrepancies in results. In studies using routine health-care data, large and unselected populations are represented in comparison to strict selection criteria for study populations in RCTs. Patients in RCTs are often younger, have less comorbidities, and show higher treatment adherence [42]. To investigate effectiveness and safety of new drugs, real-world-studies represent an important complement to RCTs [43]. In addition to methodological differences, differences of the results between our study and the RCTs might be influenced by comparing phenprocoumon to DOACs instead of warfarin. As mentioned above, phenprocoumon differs in pharmacological properties and was associated with a better TTR in previous studies as compared to warfarin. Additionally, there are great differences between the anticoagulation management in Germany and other European countries. Le Heuzey et al. compared anticoagulation management in five European countries. Two factors are distinct for Germany: Phenprocoumon is only predominant in Germany and in contrast to the other countries, INR measurements in Germany are mainly performed in physicians’ offices and as self-management, while in other countries those are also performed in hospitals, anticoagulation centres, and laboratories [22]. It is possible that both factors affect the TTR. In contrast to our results favouring phenprocoumon, Nielsen et al. did not find statistically significant differences regarding thromboembolic embolism in a real-world-study with ld-DOACs and warfarin [19]. Regarding overall bleeding risk, they showed a small benefit only for dabigatran, associated with an advantage in haemorrhagic stroke but not in major bleeding. Showing a higher risk of death in the ld-rivaroxaban and ld-apixaban cohort, our results are consistent with Nielsen et al. The small differences to the results of Nielsen et al. could hence be partially triggered by comparing ld-DOACs to warfarin instead of phenprocoumon.

Our study generates the hypothesis that ld-DOAC therapy, as practiced in Germany today, might be inferior to phenprocoumon. In the absence of an RCT with phenprocoumon, further research is needed to affirm or refute this hypothesis. Future research questions should focus on, whether the beneficial effect of phenprocoumon, as shown in our study, is triggered by an inappropriate use of ld-DOAC and/or by differences between phenprocoumon and warfarin.

### Limitations

As in any retrospective observational study using claims data, adjustments and matching could only be based on information available in the data. Therefore, residual bias driven by undocumented information cannot be ruled out. Coding of diagnoses is not perfectly accurate, especially for smoking status (in Germany frequently coded as a diagnosis (ICD-10 F17.1)) or obesity [44]. The effect of TTR cannot be calculated, as data did not provide information of the results of INR testing. The physicians’ rationales for prescribing a reduced dosage cannot be derived from the data. The effect of the TTR on the outcomes and the proportion of patients with an inadequate reduced dosage cannot be determined. As a strength of all real-world studies, the results potentially better reflect the actual health care situation by its large and more representative study population with wide selection criteria in comparison to RCTs.

## Conclusion

Our data revealed statistically significant lower rates of thromboembolic events and death for phenprocoumon without a statistically significant increase in major bleeding despite a high number of patients analysed. As a hypothesis, phenprocoumon might be superior to ld-DOAC regime as practiced today. Due to the natural limitations of real-world studies the results of our study should be evaluated by RCTs comparing ld-DOACs to phenprocoumon. As long as these RCTs don’t exist, real-world studies form the highest level of evidence available if it comes to comparing ld-DOACs with phenprocoumon. According to them, phenprocoumon might be the better choice than ld-DOACs for high-risk patients with AF.

## Supplementary Information

**Additional file 1.** PZN numbers, ICD-10- and ATC-codes, Baseline characteristics, Follow-up time, Cox-regressions analyses, Table S1-S5, Fig. S1.

## Data Availability

Because of the confidential nature of in- and outpatient claims data, a permission for public availability of the data is not possible. The permission to access the data is restricted to research and subjects to the consent of the health insurance funds.

## Abbreviations

AF: Atrial fibrillation
OAC: Oral anticoagulant
DOAC: Direct oral anticoagulant
VKA: Vitamin-K-antagonist
SmPCs: Summaries of product characteristics
EMA: European Medicines Agency
AkdÄ: Drug Commission of the German Medical Association (“Arzneimittelkommission der deutschen Ärzteschaft“)

## References

[1] Khurshid S, Choi SH, Weng LC, Wang EY, Trinquart L, Benjamin EJ (2018). Frequency of cardiac rhythm abnormalities in a half million adults. Circ Arrhythm Electrophysiol.

[2] Wolf PA, Abbott RD, Kannel WB (1991). Atrial fibrillation as an independent risk factor for stroke: the Framingham study. Stroke..

[3] Arzneimittelkommission der deutschen Ärzteschaft (AkdÄ) (2019). Orale Antikoagulation bei nicht valvulärem Vorhofflimmern Empfehlungen zum Einsatz der direkten oralen Antikoagulanzien Dabigatran, Apixaban, Edoxaban und Rivaroxaban.

[4] Hein L, Wille H, Schwabe U, Paffrath D, Ludwig W-D, Klauber J (2019). Antithrombotika und Antihämorrhagika. Arzneiverordnungs-Report 2019.

[5] Granger CB, Alexander JH, McMurray JJ, Lopes RD, Hylek EM, Hanna M (2011). Apixaban versus warfarin in patients with atrial fibrillation. N Engl J Med.

[6] Connolly SJ, Ezekowitz MD, Yusuf S, Eikelboom J, Oldgren J, Parekh A (2009). Dabigatran versus warfarin in patients with atrial fibrillation. N Engl J Med.

[7] Giugliano RP, Ruff CT, Braunwald E, Murphy SA, Wiviott SD, Halperin JL (2013). Edoxaban versus warfarin in patients with atrial fibrillation. N Engl J Med.

[8] Patel MR, Mahaffey KW, Garg J, Pan G, Singer DE, Hacke W (2011). Rivaroxaban versus warfarin in nonvalvular atrial fibrillation. N Engl J Med.

[9] European Medicines Agency (2011). Eliquis: EPAR - Product Information.

[10] European Medicines Agency (2009). Xarelto : EPAR - Product Information.

[11] European Medicines Agency (2015). Lixiana : EPAR - Product Information.

[12] European Medicines Agency (2009). Pradaxa : EPAR - Product Information.

[13] Steinberg BA, Shrader P, Pieper K, Thomas L, Allen LA, Ansell J (2018). Frequency and outcomes of reduced dose non-vitamin K antagonist anticoagulants: results from ORBIT-AF II (the outcomes registry for better informed treatment of atrial fibrillation II). J Am Heart Assoc.

[14] Hohnloser SH, Basic E, Hohmann C, Nabauer M (2018). Effectiveness and safety of non-vitamin K Oral anticoagulants in comparison to Phenprocoumon: data from 61,000 patients with atrial fibrillation. Thromb Haemost.

[15] Mueller S, Groth A, Spitzer SG, Schramm A, Pfaff A, Maywald U (2018). Real-world effectiveness and safety of oral anticoagulation strategies in atrial fibrillation: a cohort study based on a German claims dataset. Pragmat Obs Res.

[16] Zeymer U, Lober C, Wolf A, Richard F, Schäfer H, Taggeselle J (2020). Use, persistence, efficacy, and safety of Apixaban in patients with non-Valvular atrial fibrillation in unselected patients in Germany. Results of the prospective Apixaban in atrial fibrillation (APAF) registry. Cardiol Ther.

[17] Staerk L, Gerds TA, Lip GYH, Ozenne B, Bonde AN, Lamberts M (2018). Standard and reduced doses of dabigatran, rivaroxaban and apixaban for stroke prevention in atrial fibrillation: a nationwide cohort study. J Intern Med.

[18] Miyazaki M, Matsuo K, Uchiyama M, Nakamura Y, Sakamoto Y, Misaki M (2020). Inappropriate direct oral anticoagulant dosing in atrial fibrillation patients is associated with prescriptions for outpatients rather than inpatients: a single-center retrospective cohort study. J Pharm Health Care Sci.

[19] Nielsen PB, Skjoth F, Sogaard M, Kjaeldgaard JN, Lip GY, Larsen TB (2017). Effectiveness and safety of reduced dose non-vitamin K antagonist oral anticoagulants and warfarin in patients with atrial fibrillation: propensity weighted nationwide cohort study. BMJ..

[20] Beinema M, Brouwers JR, Schalekamp T, Wilffert B (2008). Pharmacogenetic differences between warfarin, acenocoumarol and phenprocoumon. Thromb Haemost.

[21] Prochaska JH, Göbel S, Keller K, Coldewey M, Ullmann A, Lamparter H (2015). Quality of oral anticoagulation with phenprocoumon in regular medical care and its potential for improvement in a telemedicine-based coagulation service–results from the prospective, multi-center, observational cohort study thrombEVAL. BMC Med.

[22] Le Heuzey JY, Ammentorp B, Darius H, De Caterina R, Schilling RJ, Schmitt J (2014). Differences among western European countries in anticoagulation management of atrial fibrillation. Data from the PREFER IN AF registry. Thromb Haemost.

[23] Swart E, Gothe H, Geyer S, Jaunzeme J, Maier B, Grobe T (2015). Gute Praxis Sekundärdatenanalyse (GPS): Leitlinien und Empfehlungen. Das Gesundheitswesen..

[24] Benchimol EI, Smeeth L, Guttmann A, Harron K, Moher D, Petersen I (2015). The REporting of studies conducted using observational routinely-collected health data (RECORD) statement. PLoS Med.

[25] Schulman S, Kearon C, Subcommittee on control of anticoagulation of the S, standardization Committee of the International Society on T, Haemostasis (2005). Definition of major bleeding in clinical investigations of antihemostatic medicinal products in non-surgical patients. J Thromb Haemost.

[26] Charlson ME, Pompei P, Ales KL, MacKenzie CR (1987). A new method of classifying prognostic comorbidity in longitudinal studies: development and validation. J Chronic Dis.

[27] Yao X, Gersh BJ, Sangaralingham LR, Kent DM, Shah ND, Abraham NS (2017). Comparison of the CHA2DS2-VASc, CHADS2, HAS-BLED, ORBIT, and ATRIA Risk Scores in Predicting Non–Vitamin K Antagonist Oral Anticoagulants-Associated Bleeding in Patients With Atrial Fibrillation. Am J Cardiol.

[28] R Core Team (2021). R: A language and environment for statistical computing. R Foundation for statistical computing Vienna, Austria.

[29] Ho D, Imai K, King G, Stuart EA (2011). MatchIt: nonparametric preprocessing for parametric causal inference. J Stat Softw.

[30] Therneau TM, Grambsch PM (2020). A package for survival analysis in R.

[31] Camm AJ, Cools F, Virdone S, Bassand JP, Fitzmaurice DA, Arthur Fox KA (2020). Mortality in patients with atrial fibrillation receiving nonrecommended doses of direct Oral anticoagulants. J Am Coll Cardiol.

[32] Steinberg BA, Shrader P, Thomas L, Ansell J, Fonarow GC, Gersh BJ (2016). Off-label dosing of non-vitamin K antagonist Oral anticoagulants and adverse outcomes: the ORBIT-AF II registry. J Am Coll Cardiol.

[33] Kang F, Ma Y, Cai A, Cheng X, Liu P, Kuang J (2021). Meta-analysis evaluating the efficacy and safety of low-intensity warfarin for patients >65 years of age with non-Valvular atrial fibrillation. Am J Cardiol.

[34] Lee KN, Choi JI, Boo KY, Kim DY, Kim YG, Oh SK (2020). Effectiveness and safety of off-label dosing of non-vitamin K antagonist anticoagulant for atrial fibrillation in Asian patients. Sci Rep.

[35] Reinecke H, Jürgensmeyer S, Engelbertz C, Gerss J, Kirchhof P, Breithardt G (2018). Design and rationale of a randomised controlled trial comparing apixaban to phenprocoumon in patients with atrial fibrillation on chronic haemodialysis: the AXADIA-AFNET 8 study. BMJ Open.

[36] Vranckx P, Valgimigli M, Eckardt L, Tijssen J, Lewalter T, Gargiulo G (2019). Edoxaban-based versus vitamin K antagonist-based antithrombotic regimen after successful coronary stenting in patients with atrial fibrillation (ENTRUST-AF PCI): a randomised, open-label, phase 3b trial. Lancet.

[37] Riesinger L, Strobl C, Leistner DM, Gori T, Akin I, Mehr M (2021). Apixaban versus PhenpRocoumon: Oral AntiCoagulation plus antiplatelet tHerapy in patients with acute coronary syndrome and atrial fibrillation (APPROACH-ACS-AF): rationale and design of the prospective randomized parallel-group, open-label, blinded-endpoint, superiority, multicenter-trial of a triple therapy versus a dual therapy in patients with atrial fibrillation and acute coronary syndrome undergoing coronary stenting. Int J Cardiol Heart Vasc.

[38] Ferner M, Wachtlin D, Konrad T, Deuster O, Meinertz T, von Bardeleben S (2016). Rationale and design of the RE-LATED AF—AFNET 7 trial: REsolution of left atrial-appendage Thrombus—effects of dabigatran in patients with atrial fibrillation. Clin Res Cardiol.

[39] Di Biase L, Callans D, Hæusler KG, Hindricks G, Al-Khalidi H, Mont L (2017). Rationale and design of AXAFA-AFNET 5: an investigator-initiated, randomized, open, blinded outcome assessment, multi-Centre trial to comparing continuous apixaban to vitamin K antagonists in patients undergoing atrial fibrillation catheter ablation. Europace..

[40] Mantha S, Ansell J (2015). Indirect comparison of dabigatran, rivaroxaban, apixaban and edoxaban for the treatment of acute venous thromboembolism. J Thromb Thrombolysis.

[41] Ufer M (2010). Comparative efficacy and safety of the novel oral anticoagulants dabigatran, rivaroxaban and apixaban in preclinical and clinical development. Thromb Haemost.

[42] Freemantle N, Marston L, Walters K, Wood J, Reynolds MR, Petersen I (2013). Making inferences on treatment effects from real world data: propensity scores, confounding by indication, and other perils for the unwary in observational research. BMJ.

[43] Camm AJ, Fox KAA (2018). Strengths and weaknesses of 'real-world' studies involving non-vitamin K antagonist oral anticoagulants. Open Heart.

[44] Angelow A, Reber KC, Schmidt CO, Baumeister SE, Chenot J-F (2019). Untersuchung der Prävalenz kardiologischer Risikofaktoren in der Allgemeinbevölkerung: Ein Vergleich ambulanter ärztlicher Abrechnungsdaten mit Daten einer populationsbasierten Studie. Das Gesundheitswesen.

