# Supplementary material for: Vitamin-K-antagonist phenprocoumon versus low-dose direct oral anticoagulants (DOACs) in patients with atrial fibrillation: a real-world analysis of German claims data
Source: Thromb J. 2022 May 26;20:31. doi: 10.1186/s12959-022-00389-9 (PMC9137171; doi:10.1186/s12959-022-00389-9)
Supplement: Supplementary file 1 — Additional file 1. PZN numbers, ICD-10- and ATC-codes, Baseline characteristics, Follow-up time, Cox-regressions analyses, Table S1-S5, Fig. S1. [file 12959_2022_389_MOESM1_ESM.pdf]

Additional Files

Table S1: PZN numbers (Pharmacy central number) of low-dose DOACs 2

Table S2: International Classification of Diseases version 10 (ICD-10)-codes for effectiveness and safety outcomes. 2

Table S3: International Classification of Diseases version 10 (ICD-10)-codes and Anatomical Therapeutic Chemical (ATC)-codes of covariates for Cox regression analysis and propensity score matching. 3

Table S4: Baseline characteristics of phenprocoumon, low-dose DOAC (ld-DOAC) cohort and subgroups (apixaban, dabigatran, edoxaban and rivaroxaban cohort) after propensity score matching. Standard mean differences (SMD), sample sizes after matching and number of unmatched datasets. 4

Table S5: Mean-follow up time by cohort and outcome. 8

Fig. S1a: Cox-Regression analysis for low-dose DOAC vs. phenprocoumon regarding death 9

Fig. S1b: Cox-Regression analysis for low-dose DOAC vs. phenprocoumon regarding thromboembolic events 10

Fig. S1c: Cox-Regression analysis for low-dose DOAC vs. phenprocoumon regarding bleeding .. 11

Table S1: PZN numbers (Pharmacy central number) of low-dose DOACs

**B01AF02 Apixaban**
3643804, 8400029, 8400035, 8400041, 10250465, 11341537, 11376429, 11376435, 13578924, 4712163, 4712186, 8400012
**B01AE07 Dabigatran**
6115862, 3420607, 3420613, 6312284, 6561863, 11009889, 11027491, 11291087, 11690769, 12607931, 12671917, 12868666, 13232025, 14015620, 9707095, 3420754, 3420760, 6561892, 6561900, 6561917, 9228199, 9328156, 10183585, 10218349, 10218355, 10261210, 10288120, 10339484, 10339509, 10339521, 10402596, 10402604, 10402685, 10737084, 10783204, 11127442, 11130183, 11130378, 11296280, 11296305, 11309114, 11341916, 11341922, 12448881, 12615296, 12638096, 12639109, 12801320, 13504647, 13659143, 13894045, 13895375, 13947511, 14015637, 14155841, 14441742, 14445881, 10218361, 10251016, 10251022, 10390829, 10944541
**B01AF03 Edoxaban**
10713994, 10714002, 10714031, 10714060, 10714083, 10714143, 10714189, 12749950, 12749967, 13695860
**B01AF01 Rivaroxaban**
8461261, 8461290, 8717186, 9647915, 9676408, 12590136, 13902371, 14406467, 14406473, 14445591, 2088536, 5459513, 5748766, 5995074, 5995080, 6410420, 6454481, 7572633, 7572662, 7610606, 7799012, 7799029, 9154791, 9721534, 9777888, 9941276, 10339455, 10381894, 10381902, 10402662, 10743771, 10764520, 10852626, 10852632, 11565001, 11898174, 12636016, 13902388, 14227440, 14254247, 14440814, 14447816, 15204369, 7536850, 7536927, 11617270, 11864962, 12433508, 13331135, 13502499, 13711866, 13721818, 13902394, 13902862, 14140147, 14336206, 10101682, 10102144, 10132139, 4369423, 4369452, 4369475, 7089598, 7605019, 8461344, 8461350, 8461367, 8461373, 8461404, 9724515, 9724521, 9724538, 9941282, 10005926, 10012139, 10012145, 10012151, 10012168, 10058590, 10058609, 10072093, 10072101, 10200906, 10200912, 10297679, 10381919, 10381925, 10381931, 10381948, 10393650, 10393667, 10393696, 10743794, 10762403, 10852649, 10852655, 10852661, 10852678, 10853560, 10948970, 10948987, 10964153, 10964176, 10999312, 10999329, 10999335, 11015708, 11096606, 11559348, 11565018, 12407801, 14334348, 11724729

Table S2: International Classification of Diseases version 10 (ICD-10)-codes for effectiveness and safety outcomes.

**ICD-codes for efficiency and safety outcomes**	
Cerebral infarction	I63
Stroke, not specified	I64
Transient cerebral ischaemic attacks	G45
Acute vascular disorders of intestine	K55.0
Bleedings in critical areas or organs	H35.6, H43.1, I60, I61, I62, G95.10, K66.1
Bleedings leading to blood transfusion (inpatient billing code 8-800)	D68.3, D69.9, I85.0, I98.3, J94.2, K22.6, K22.81, K25.0, K25.2, K25.4, K25.6, K26.0, K26.2, K26.4, K26.6, K27.0, K27.2, K27.4, K27.6, K28.0, K28.2, K28.4, K28.6, K29.0, K31.82, K55.22, K57.01, K57.11, K57.13, K57.21, K57.31, K57.33, K57.41, K57.51, K57.53, K57.81, K57.91, K57.93, K62.5, K92.2, K92.0, K92.1, N02.0, N02.1, N02.2, N02.3, N02.4, N02.5, N02.6, N02.7, N02.8, N02.9, N42.1, N95.0, R04, R31, R58

Table S3: International Classification of Diseases version 10 (ICD-10)-codes and Anatomical Therapeutic Chemical (ATC)-codes of covariates for Cox regression analysis and propensity score matching.

**Codes for covariables for Cox regression and propensity score matching**	
**Prescriptions**	**ATC-code**
Antiarrhythmic medication	C08, C01B, C07AB, C07AG
Antihypertensive medication	C02, C06A
Antiplatelet therapy	B01AC
Antipsychotic medication	N05A
Anti-ulcer therapy	A02B
Cardiac glycosides	C01A
Diuretics	C03
Heparin	B01AB
Lipid lowering medication	C10
NSAIDs	M01
Oral corticosteroids	H02
**Comorbidities**	**ICD-codes**
Adiposity	E66
Alcohol abuse	F10
Arterial hypertension	I10, I15
Atherosclerosis	I70
Cachexia	R64
Cancer	C00-C97
Dementia	F00, F01, F02, F03
Diabetes	E10-E14
Liver disease	K70-K77, B15-B19
Myocardial infarction	I21, I22
Nicotine abuse	Z72.2, F17
Orthopaedic implant	Z96.64, Z96.65, T84.04, T84.05
Renal impairment	N18, N19, N17, I12.0, I13.1, I13.2, O08.4, Z99.2, P96.0, Z49.1, N99.0, O90.4, T79.5
- Acute renal impairment	N17
- Moderate chronic impairment	N18.1, N18.2, N18.3
- Severe chronic impairment	N18.4, N18.5
Stroke	I64
Thrombosis	I80-I82

Table S4: Baseline characteristics of phenprocoumon, low-dose DOAC (ld-DOAC) cohort and subgroups (apixaban, dabigatran, edoxaban and rivaroxaban cohort) after propensity score matching. Standard mean differences (SMD), sample sizes after matching and number of unmatched datasets.

**After Matching**	**Phenprocoumon**	**ld-DOAC**	**SMD**	
n	14,818	14,818		
n (unmatched)	5,361	6,906		
Distance	0.51	0.53	0.08	
Baseline characteristics (mean ± SD):				
Age	76.96 (8.91)	77.49 (10.12)	0.05	
Female persons (%)	44.95	45.69	0.01	
CCI = 0	7.87	7.44	-0.02	
CCI ≥ 5	26.16	27.39	0.03	
CCI = 1-2	33.04	32.39	-0.01	
CCI = 3-4	32.93	32.78	0.00	
CHA_2DS_2-VASc-Score (without gender)	3.93 (1.52)	3.99 (1.58)	0.04	
Prescriptions in addition to OAC (%):				
≤ 4	12.79	12.26		
5-9	38.73	37.89	-0.02	
10 -14	30.04	30.22	0.00	
≥ 15	18.44	19.62	0.03	
Comorbidities: Proportion of patients with … (%)				
Acute renal impairment	7.38	8.04	0.02	
Moderate chronic renal impairment	25.97	27.39	0.03	
Severe chronic renal impairment	6.38	6.55	0.01	
Renal impairment (total)	38.27	40.13	0.04	
Dementia	10.78	11.74	0.02	
Thrombosis	3.91	3.80	-0.01	
Arterial hypertension	90.31	90.38	0.00	
Diabetes	38.37	38.78	0.01	
Nicotine abuse	7.77	7.65	0.00	
Alcohol abuse	3.35	3.24	-0.01	
Myocardial infarction	10.11	10.63	0.02	
Stroke	4.51	4.74	0.01	
Atherosclerosis	20.72	20.75	0.00	
Cancer	23.12	23.51	0.01	
Liver disease	18.25	18.35	0.00	
Cachexia	0.90	1.13	0.02	
Adiposity	26.44	26.09	-0.01	
Orthopaedic implant	4.41	5.16	0.03	
Comedication: Proportion of patients with … (%)				
Antihypertensive medication	7.59	7.88	0.01	

Heparin	13.98	13.73	-0.01			
Diuretics	56.82	57.52	0.01			
Antiarrhythmic medication	87.32	87.54	0.01			
NSAIDs	32.74	32.85	0.00			
Antiplatelet therapy	29.62	31.16	0.03			
Lipid lowering medication	48.08	48.70	0.01			
Anti-ulcer therapy	49.24	50.18	0.02			
Cardiac glycosides	10.84	10.46	-0.01			
Oral corticosteroids	13.29	13.65	0.01			
Antipsychotic medication	5.47	5.96	0.02			
Inpatient diagnosis before index date … (%)						
Thromboembolic event	7.18	8.08	0.03			
Bleeding	2.55	2.68	0.01			
Outpatient diagnosis before index date … (%)						
Thromboembolic event	11.16	11.95	0.02			
Bleeding (no blood transfusion)	1.72	1.77	0.00			
**After Matching**	**Phenprocoumon**	**ld-apixaban**	**SMD**	**Phenprocoumon**	**ld-dabigatran**	**SMD**
n	8,991	8,991		1,908	1,908	
n (unmatched)	11,188	2,006		18,271	6	
Distance	0.42	0.43	0.06	0.14	0.14	0.00
Baseline characteristics (mean ± SD):						
Age	79.78 (7.94)	80.21 (8.76)	0.05	76.37 (9.64)	76.46 (10.35)	0.01
Female persons	50.06	50.19	0.00	42.61	43.71	0.02
CCI = 0	5.58	5.28	-0.01	8.54	7.97	-0.02
CCI ≥ 5	31.14	31.46	0.01	26.57	25.16	-0.03
CCI = 1-2	29.45	29.11	-0.01	33.70	33.75	0.00
CCI = 3-4	33.82	34.15	0.01	31.18	33.12	0.04
CHA_2DS_2-VASc-Score (without gender)	4.23 (1.48)	4.26 (1.49)	0.02	4.09 (1.64)	4.05 (1.65)	-0.02
Prescriptions in addition to OAC (%):						
≤ 4	10.33	10.17		14.88	13.68	
5-9	35.85	35.65	0.00	35.17	38.57	0.07
10 -14	32.11	32.29	0.00	32.02	30.87	-0.02
≥ 15	21.71	21.90	0.00	17.92	16.88	-0.03
Comorbidities: Proportion of patients with … (%)						
Acute renal impairment	9.51	9.94	0.01	5.56	4.98	-0.03
Moderate chronic renal impairment	31.32	32.04	0.02	22.75	21.07	-0.04
Severe chronic renal impairment	8.10	7.91	-0.01	1.62	1.57	0.00
Renal impairment	45.69	46.06	0.01	32.08	29.87	-0.05
Dementia	15.26	16.59	0.03	13.05	12.84	-0.01
Thrombosis	3.81	3.80	0.00	2.88	2.73	-0.01

Arterial hypertension	91.42	91.71	0.01	89.83	89.88	0.00
Diabetes	39.78	40.00	0.00	35.22	35.43	0.00
Nicotine abuse	6.76	6.47	-0.01	8.12	8.18	0.00
Alcohol abuse	3.00	2.94	0.00	3.09	3.20	0.01
Myocardial infarction	10.33	10.51	0.01	14.26	13.47	-0.02
Stroke	5.13	5.33	0.01	8.02	7.81	-0.01
Atherosclerosis	21.89	22.01	0.00	20.07	20.39	0.01
Cancer	25.03	25.19	0.00	22.69	21.80	-0.02
Liver disease	18.07	17.74	-0.01	16.67	17.35	0.02
Cachexia	1.28	1.35	0.00	0.73	0.84	0.01
Adiposity	24.85	24.49	-0.01	22.85	22.22	-0.02
Orthopaedic implant	5.99	6.14	0.01	4.66	5.03	0.02
Comedication: Proportion of patients with … (%)						
Antihypertensive medication	8.41	8.14	-0.01	7.29	6.76	-0.02
Heparin	11.40	11.73	0.01	10.06	9.70	-0.01
Diuretics	62.06	61.93	0.00	49.21	48.69	-0.01
Antiarrhythmic medication	87.81	87.71	0.00	85.32	85.22	0.00
NSAIDs	32.10	31.65	-0.01	35.38	34.75	-0.01
Antiplatelet therapy	32.71	33.29	0.01	37.16	36.16	-0.02
Lipid lowering medication	47.94	48.07	0.00	56.08	53.98	-0.04
Anti-ulcer therapy	52.51	53.03	0.01	48.74	48.85	0.00
Cardiac glycosides	11.11	10.86	-0.01	7.60	7.97	0.01
Oral corticosteroids	14.23	14.17	0.00	14.31	14.36	0.00
Antipsychotic medication	7.18	7.97	0.03	7.97	7.49	-0.02
Inpatient diagnosis before index date … (%)						
Thromboembolic event	9.38	10.18	0.02	20.60	20.55	0.00
Bleeding	3.13	3.21	0.00	3.09	3.09	0.00
Outpatient diagnosis before index date … (%)						
Thromboembolic event	12.82	12.94	0.00	19.29	19.55	0.01
Bleeding (no blood transfusion)	2.21	2.19	0.00	1.83	1.94	0.01
**After Matching**	**Phenprocoumon**	**ld-edoxaban**	**SMD**	**Phenprocoumon**	**ld-rivaroxaban**	**SMD**
n	2,235	2,235		6,478	6,478	
n (unmatched)	17,944	20		13,701	80	
Distance	0.17	0.17	0.01	0.30	0.31	0.02
Baseline characteristics (mean ± SD):						
Age	80.02 (8.45)	79.87 (9.17)	-0.02	76.72 (9.48)	76.88 (10.36)	0.02
Female persons	52.30	53.15	0.02	44.09	44.32	0.00
CCI = 0	6.49	6.13	-0.01	7.26	7.32	0.00
CCI ≥ 5	31.05	30.25	-0.02	29.01	28.50	-0.01
CCI = 1-2	29.75	29.53	0.00	30.27	30.67	0.01
CCI = 3-4	32.71	34.09	0.03	33.47	33.51	0.00

CHA_2DS_2-VASc-Score (without gender)	4.05 (1.49)	4.05 (1.53)	0.00	3.98 (1.58)	3.97 (1.61)	-0.01
Prescriptions in addition to OAC (%):						
≤ 4	12.48	11.99		11.52	11.36	
5-9	36.60	37.90	0.03	36.23	35.54	-0.01
10 -14	30.25	29.49	-0.02	30.84	31.35	0.01
≥ 15	20.67	20.63	0.00	21.41	21.75	0.01
Comorbidities: Proportion of patients with … (%)						
Acute renal impairment	9.93	9.44	-0.02	8.03	7.90	0.00
Moderate chronic renal impairment	36.11	36.29	0.00	29.96	29.55	-0.01
Severe chronic renal impairment	7.65	7.29	-0.01	5.20	4.79	-0.02
Renal failure	49.57	49.13	-0.01	41.82	41.77	0.00
Dementia	15.97	16.20	0.01	13.68	14.02	0.01
Thrombosis	3.94	3.13	-0.05	4.85	4.69	-0.01
Arterial hypertension	89.26	89.71	0.01	90.14	90.29	0.01
Diabetes	39.91	39.96	0.00	41.62	40.74	-0.02
Nicotine abuse	7.11	6.17	-0.04	9.11	8.57	-0.02
Alcohol abuse	3.67	3.40	-0.01	3.83	3.86	0.00
Myocardial infarction	7.11	7.87	0.03	14.14	14.28	0.00
Stroke	4.47	4.03	-0.02	4.43	4.34	0.00
Atherosclerosis	20.76	21.52	0.02	21.30	21.15	0.00
Cancer	26.35	26.00	-0.01	23.34	23.17	0.00
Liver disease	20.36	20.09	-0.01	18.42	18.26	0.00
Cachexia	1.79	1.70	-0.01	1.30	1.53	0.02
Adiposity	23.85	23.80	0.00	27.51	27.25	-0.01
Orthopaedic implant	10.43	10.87	0.01	6.95	7.89	0.03
Comedication: Proportion of patients with … (%)						
Antihypertensive medication	9.17	8.95	-0.01	7.22	7.36	0.01
Heparin	10.69	10.47	-0.01	9.76	10.59	0.03
Diuretics	57.76	57.18	-0.01	56.00	56.10	0.00
Antiarrhythmic medication	85.41	85.73	0.01	86.91	86.89	0.00
NSAIDs	31.77	31.10	-0.01	34.47	34.93	0.01
Antiplatelet therapy	28.72	30.51	0.04	38.70	38.76	0.00
Lipid lowering medication	43.58	45.23	0.03	48.44	48.58	0.00
Anti-ulcer therapy	48.28	49.35	0.02	52.61	53.52	0.02
Cardiac glycosides	9.13	8.72	-0.01	9.69	9.54	-0.01
Oral corticosteroids	14.59	13.91	-0.02	14.68	14.80	0.00
Antipsychotic medication	9.17	8.23	-0.03	7.05	7.38	0.01
Inpatient diagnosis before index date … (%)						
Thromboembolic event	6.85	7.16	0.01	7.21	6.92	-0.01
Bleeding	2.64	2.06	-0.04	2.59	2.47	-0.01

Outpatient diagnosis before index date … (%)						
Thromboembolic event	11.23	11.10	0.00	10.91	11.07	0.00
Bleeding (no blood transfusion)	1.79	1.92	0.01	1.90	1.70	-0.02

Table S5: Mean-follow up time by cohort and outcome.

Mean follow-up time (in days)	Phenprocoumon vs. low-dose DOAC			
	VKA	DOAC		
N	14,818	14,818		
Thromboembolic events	310.68	277.36		
Deceased	313.93	281.14		
Bleeding	309.09	276.64		
**Mean follow-up time (in days)**	**Phenprocoumon vs. ld-apixaban**		**Phenprocoumon vs. ld-dabigatran**	
	**VKA**	**DOAC**	**VKA**	**DOAC**
N	8,991	8,991	1,908	1,908
Thromboembolic events	304.24	283.45	308.31	281.27
Deceased	307.90	288.16	311.98	285.15
Bleeding	302.41	283.71	307.57	281.77
**Mean follow-up time (in days)**	**Phenprocoumon vs. ld-edoxaban**		**Phenprocoumon vs. ld-rivaroxaban**	
	**VKA**	**DOAC**	**VKA**	**DOAC**
N	2,235	2,235	6,478	6,478
Thromboembolic events	302.73	284.73	306.54	267.20
Deceased	305.96	288.40	309.62	270.06
Bleeding	301.06	283.84	304.73	264.58

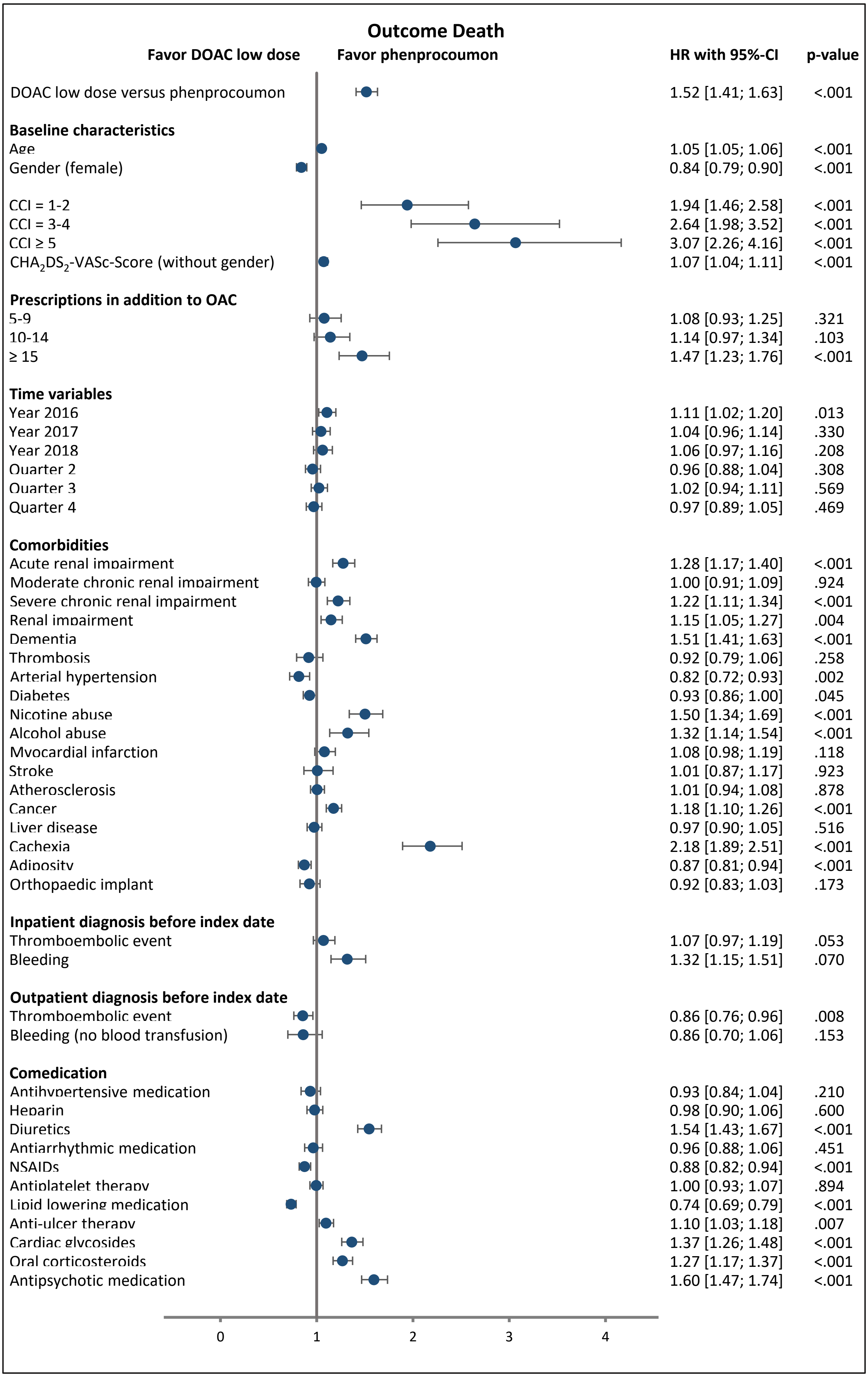

Fig. S1a: Cox-Regression analysis for low-dose DOAC vs. phenprocoumon regarding death

Outcome Thromboembolic Events

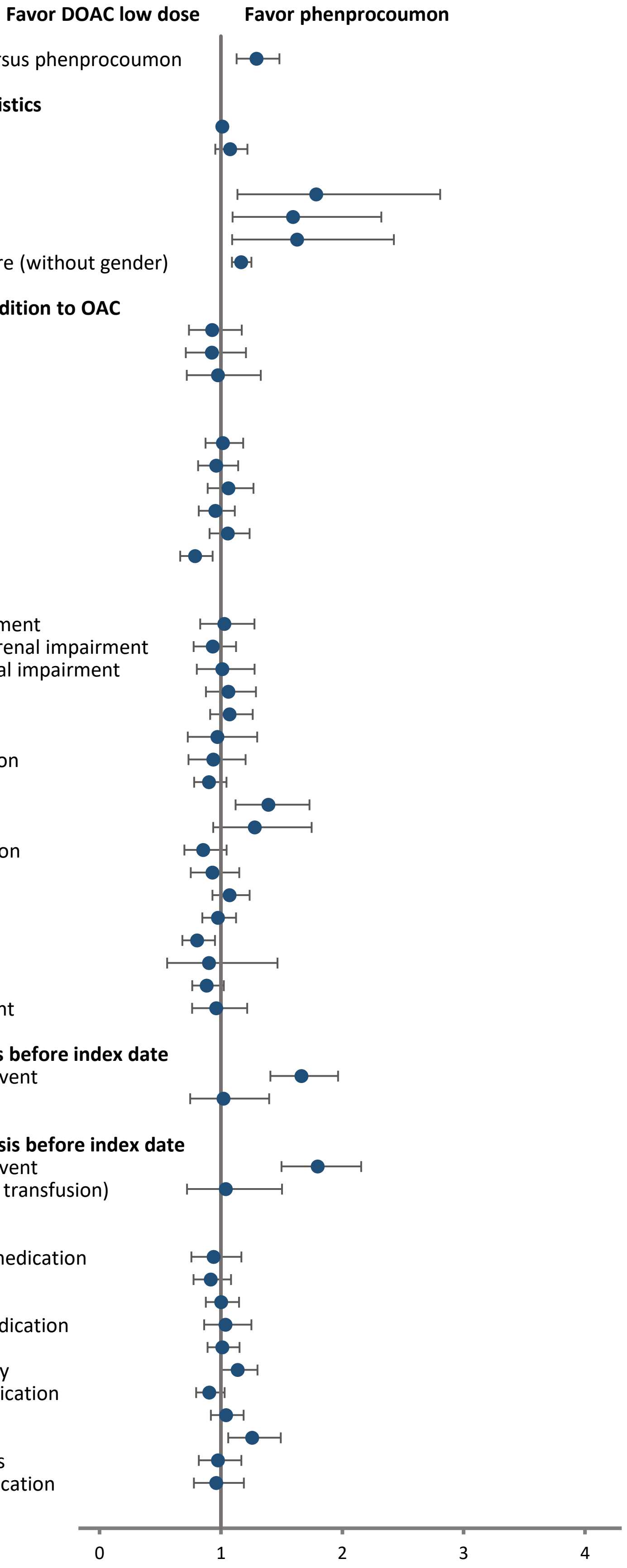

	HR with 95%-CI	p-value
DOAC low dose versus phenprocoumon	1.29 [1.13; 1.48]	<.001
**Baseline characteristics**		
Age	1.01 [1.00; 1.02]	.003
Gender (female)	1.08 [0.95; 1.22]	.229
CCI ≥ 5	1.79 [1.14; 2.81]	.012
CCI = 1-2	1.60 [1.10; 2.32]	.015
CCI = 3-4	1.63 [1.09; 2.43]	.017
CHA_2DS_2-VASc-Score (without gender)	1.17 [1.09; 1.25]	<.001
**Prescriptions in addition to OAC**		
5-9	0.93 [0.74; 1.17]	.535
10-14	0.93 [0.71; 1.21]	.569
≥ 15	0.98 [0.72; 1.33]	.883
**Time variables**		
Year 2016	1.02 [0.87; 1.18]	.830
Year 2017	0.96 [0.81; 1.14]	.667
Year 2018	1.06 [0.89; 1.27]	.497
Quarter 2	0.95 [0.82; 1.11]	.558
Quarter 3	1.06 [0.91; 1.24]	.471
Quarter 4	0.79 [0.66; 0.93]	.005
**Comorbidities**		
Acute renal impairment	1.03 [0.83; 1.28]	.795
Moderate chronic renal impairment	0.93 [0.78; 1.12]	.471
Severe chronic renal impairment	1.01 [0.80; 1.28]	.921
Renal impairment	1.06 [0.88; 1.29]	.535
Dementia	1.07 [0.91; 1.26]	.399
Thrombosis	0.97 [0.73; 1.30]	.847
Arterial hypertension	0.94 [0.73; 1.20]	.620
Diabetes	0.90 [0.78; 1.05]	.173
Nicotine abuse	1.39 [1.12; 1.73]	.003
Alcohol abuse	1.28 [0.94; 1.75]	.121
Myocardial infarction	0.86 [0.70; 1.05]	.130
Stroke	0.93 [0.75; 1.15]	.506
Atherosclerosis	1.07 [0.93; 1.24]	.334
Cancer	0.98 [0.85; 1.12]	.737
Liver disease	0.81 [0.68; 0.95]	.011
Cachexia	0.90 [0.56; 1.47]	.682
Adiposity	0.88 [0.76; 1.02]	.100
Orthopaedic implant	0.96 [0.76; 1.22]	.754
**Inpatient diagnosis before index date**		
Thromboembolic event	1.66 [1.41; 1.97]	<.001
Bleeding	1.02 [0.75; 1.40]	.893
**Outpatient diagnosis before index date**		
Thromboembolic event	1.80 [1.50; 2.16]	<.001
Bleeding (no blood transfusion)	1.04 [0.72; 1.50]	.830
**Comedication**		
Antihypertensive medication	0.94 [0.76; 1.17]	.580
Heparin	0.92 [0.77; 1.08]	.306
Diuretics	1.00 [0.88; 1.15]	.961
Antiarrhythmic medication	1.04 [0.86; 1.25]	.690
NSAIDs	1.01 [0.89; 1.15]	.843
Antiplatelet therapy	1.14 [1.00; 1.30]	.057
Lipid lowering medication	0.91 [0.80; 1.03]	.133
Anti-ulcer therapy	1.04 [0.92; 1.19]	.516
Cardiac glycosides	1.26 [1.06; 1.49]	.009
Oral corticosteroids	0.98 [0.82; 1.17]	.804
Antipsychotic medication	0.96 [0.78; 1.19]	.718

Fig. S1b: Cox-Regression analysis for low-dose DOAC vs. phenprocoumon regarding thromboembolic events

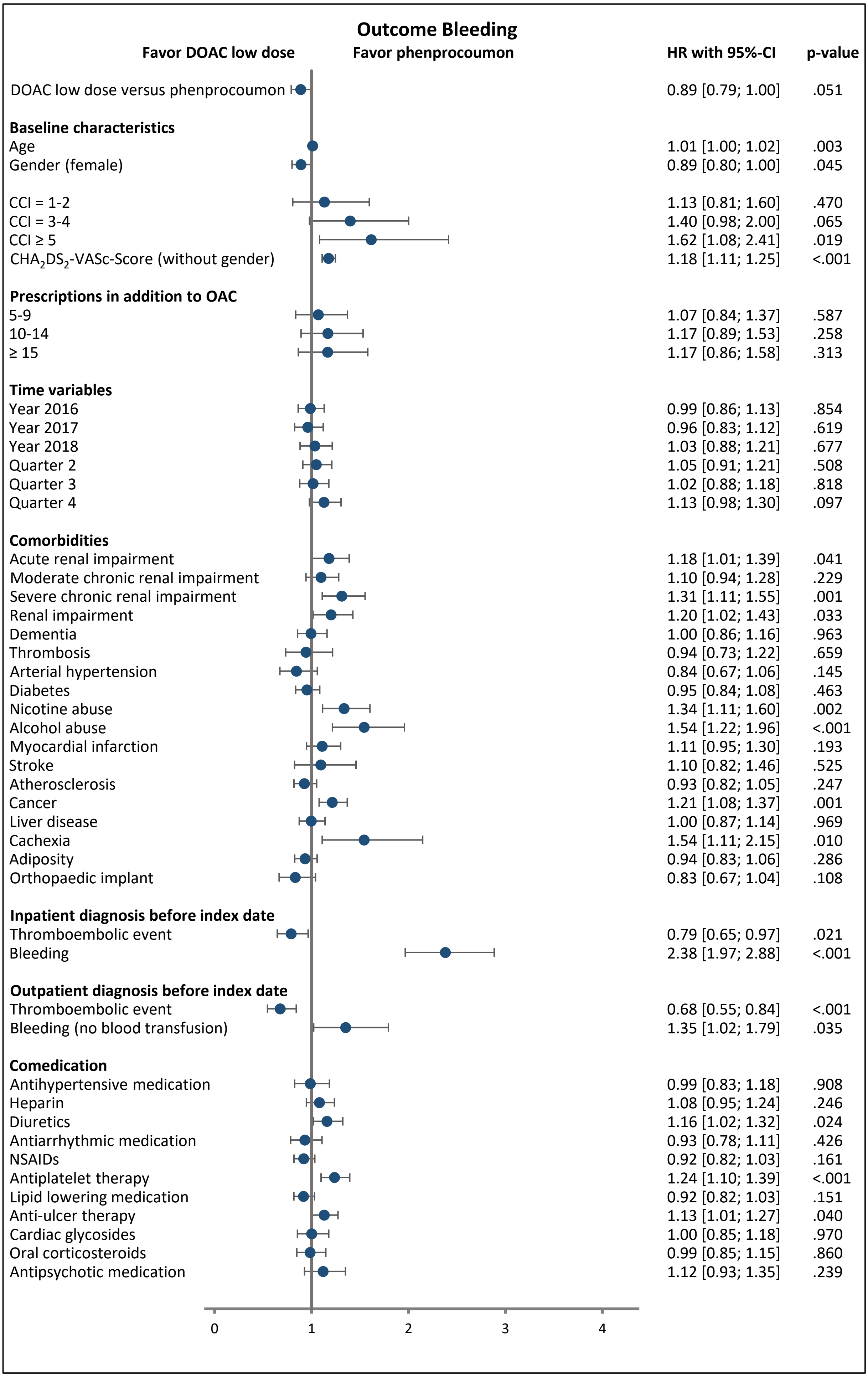

Fig. S1c: Cox-Regression analysis for low-dose DOAC vs. phenprocoumon regarding bleeding
